# *Urtica dioica* L. Alleviates Bisphenol A-Induced Testicular Toxicity in Rats via Modulation of Oxidative Stress, Inflammation, and Apoptosis

**DOI:** 10.3390/vetsci13080840

**Published:** 2026-08-20

**Authors:** Seydi Ahmet Şengül, Gülsüm Pektanç Şengül, Fırat Aşır, Nurdan Coşkun, Irmak İçen Taşkın, Oğuz Kaan Yalçın

**Affiliations:** 1 Department of Pharmacology and Toxicology, Faculty of Veterinary Medicine, Hatay Mustafa Kemal University, 31060 Hatay, Türkiye; 2 Technology and Research & Development Center (MARGEM), Hatay Mustafa Kemal University, 31060 Hatay, Türkiye; gulsumpektanc@mku.edu.tr; 3 Department of Histology and Embryology, Medical Faculty, Dicle University, 21280 Diyarbakır, Türkiye; firatasir@gmail.com; 4 Department of Reproduction and Artificial Insemination, Faculty of Veterinary Medicine, Hatay Mustafa Kemal University, 31060 Hatay, Türkiye; nurdancoskun@mku.edu.tr (N.C.); oguzkaan.yalcin@mku.edu.tr (O.K.Y.); 5 Department of Molecular Biology and Genetics, Faculty of Science and Art, Inonu University, 44280 Malatya, Türkiye; irmak.taskin@inonu.edu.tr

**Keywords:** apoptosis, bisphenol A, inflammation, oxidative stress, testicular toxicity, *Urtica dioica* L.

## Abstract

In this study, the aim was to investigate the effect of *Urtica dioica* L. (UD) on bisphenol A (BPA)-induced testicular toxicity in rats. Forty-eight male rats were divided into six groups: control, BPA (50 mg/kg), UD100 (100 mg/kg), UD200 (200 mg/kg), BPA + UD100, and BPA + UD200. UD ameliorated the BPA-induced reductions in sperm concentration and motility, membrane integrity and decreased increments in abnormalities of sperm. The results showed that BPA exposure significantly decreased testosterone levels, and moreover, co-administration of UD appeared to enhance this effect. Treatment with UD alleviated BPA-induced testicular apoptosis by attenuation of Bax and cleaved caspase-3 levels by increasing Bcl-2 level. Moreover, it was significantly reduced inflammation by decreasing the expression of TNF-α, IL-1ß, and IL-6 in the testis tissue compared to the BPA group. It was also determined that BPA-induced histopathological damage was significantly mitigated by UD co-administration. In the BPA group, CAT, SOD, and GPx levels decreased compared with those of the control group. However, co-administration of UD showed an ameliorative effect on the other antioxidant enzymes except for SOD. These findings revealed that UD can be used as a potential and effective agent to ameliorate BPA-induced testicular toxicity due to its antioxidant, anti-inflammatory and antiapoptotic properties.

## Simple Summary

Exposure to food contaminants is considered one of the significant environmental risk factors that play a role in the etiology of male infertility. Bisphenol A (BPA), which is widely found in the composition of plastic products, holds a significant place among foodborne contaminants. Plants with high antioxidant capacity are frequently used for their protective effects against the toxic effects caused by BPA, which can act as an endocrine-disrupting chemical. The aim of this study was to evaluate the protective and ameliorative effects of *Urtica dioica* L. (UD) against BPA-induced testicular dysfunction. The findings indicate that UD treatment significantly reduced the adverse effects of BPA on the male reproductive system through antioxidant, anti-inflammatory, and antiapoptotic mechanisms. These findings suggest that UD could be considered a potential pharmacological agent in the treatment of infertility caused by BPA exposure.

## 1. Introduction

Bisphenol A (BPA), one of the most widely produced chemicals worldwide, is used as a key component of epoxy resins to coat the inner surface of metallic food and beverage cans, as well as to form polycarbonate plastics used in optics, medical packaging, construction materials, household items, baby bottles and recyclable water bottles [1,2]. BPA penetrates the body through inhalation, digestion, and skin and exhibits antiandrogenic and estrogenic properties [3,4]. BPA, which is an antagonist of androgen and estrogen receptors, affects hormone-receptor binding functions, causing changes in reproductive organ weight, decreased sperm motility and dysfunction in gene expression levels related to male fertility [5,6]. Mitochondria, one of the organelles of the male reproductive system, have a very important role in epigenetic regulation, cell differentiation, testosterone production and spermatogenesis in the testes [7]. However, increased reactive oxygen species (ROS) levels as a result of BPA exposure cause disorders in the mitochondrial antioxidant system, leading to cell apoptosis and oxidative stress. Affecting testicular cells such as germ, Leydig, and Sertoli cells, BPA may cause the formation of abnormal testicular function by disrupting hormonal homeostasis. As a result of the dysfunction it creates in the Leydig cells that produce sex hormones, it causes irregularities in genes associated with male fertility, abnormal male phenotypes, and male infertility as a result of hormonal disorders [8].

Herbal products are widely used in the treatment of male infertility [9]. In recent years, the increasing trend toward the use of plants for prophylactic and therapeutic purposes has made *Urtica dioica* L. (UD) to the center of attention in this regard [10]. UD, belonging to the Urticaceae plant family, is a perineal herbaceous plant commonly known as nettle [11]. UD has a phytochemical profile rich in carotenoids, chlorophylls, vitamins, proteins, minerals, flavonoids, and phenolic compounds [12]. UD, which has been traditionally used in many regions of the world to treat various diseases, has been reported to exhibit strong antioxidant activity by neutralizing ROS, in addition to its anti-inflammatory, antiproliferative, and anticancer effects [13]. Chlorogenic acid, one of the major phenolic components of UD, has been shown to reduce tissue damage and promote healing in experimental models of testicular injury due to its antioxidant and anti-inflammatory effects [14]. In contrast, direct evidence regarding the protective effect of quinic acid on testicular injury is limited. The fact that research has largely focused on chlorogenic acid, since it is an ester of caffeic acid, and quinic acid may partially explain the limited literature on quinic acid [15]. Nevertheless, it has been reported that quinic acid reduces oxidative stress and suppresses the inflammatory response in various experimental models of tissue damage [16]. Accordingly, in BPA-induced testicular damage where oxidative stress and inflammation are among the key pathological mechanisms it is thought that quinic acid may contribute to protective effects alongside chlorogenic acid and other phenolic compounds. Therefore, it is assessed that the pharmacological effects of UD may stem from the synergistic or complementary effects of the bioactive compounds it contains, primarily chlorogenic acid and quinic acid.

Medicinal plants play a significant role not only in the prevention of diseases under conditions of oxidative damage but also in their treatment [17]. It is also known to have testicular protective effects by improving sperm parameters and spermatozoa quality [18]. Despite the many pharmacological properties of UD, further details are required to determine its efficacy against BPA-induced testicular toxicity. For this reason, we aimed to use UD, which has a high antioxidant capacity, as a preservative and treatment against apoptosis, inflammation, and tissue damage due to oxidative stress caused by BPA in this study. A deeper understanding of UD’s protective role could help in devising novel treatments for BPA-associated testicular damage.

## 2. Materials and Methods

### 2.1. Preparation of UD Hydroalcoholic Extract

Fresh aerial parts of UD were collected from Hatay Province, Türkiye, authenticated by Prof. Dr. Ilhan Uremiş, and deposited in the Herbarium of the Centre for Implementation and Research of Plant Health Clinic, Hatay Mustafa Kemal University, under voucher specimen number MKUBK-H-0296. The freshly collected plant material was washed several times with distilled water, air-dried, and finely ground into powder using a laboratory blender (Bosch, Stuttgart, Germany). A total of 100 g of powdered plant material was macerated with 1000 mL of a 70% ethanol-distilled water mixture for 72 h at room temperature, protected from light. Then, the mixture was filtered using Whatman No. 44 filter paper (Macherey-Nagel, Düren, Germany). The solvent was removed under reduced pressure using a rotary evaporator at 40 °C (IKA RV 3 V Flex, IKA, Staufen, Germany). The residue was lyophilized for 24 h at −50 °C and 10^–3^ mbar under vacuum using a freeze-dryer (Teknosem, Toros TRS 2/2 V, Tuzla, Türkiye) and stored at −20 °C until use [19]. The extraction yield was 5.7%.

### 2.2. Determination of the Total Phenolic Content (TPC) in UD Extract

The TPC of UD extract was quantified using the Folin–Ciocalteu colorimetric assay according to the method described by Singleton et al. [20], with gallic acid (Sigma-Aldrich, St. Louis, MO, USA) used as the reference standard. For the analysis, 100 μL of the ethanolic extract solution was combined with 1 mL of 10% Folin–Ciocalteu’s phenol reagent (Merck, Darmstadt, Germany), followed by the addition of 2.5 mL of 10% sodium carbonate (Na_2_CO_3_) solution (Merck, Darmstadt, Germany). The reaction mixture was incubated at room temperature in the dark for 1 h before measurement. Absorbance was recorded at 765 nm using an Ultraviolet–visible (UV-Vis) spectrophotometer (Jasco, Tokyo, Japan). TPC was calculated from a calibration curve prepared with ethanolic gallic acid solutions at concentrations of 25, 50, 100, 200, and 400 μg/mL. The TPC was measured as gallic acid equivalents (mg of GAE/g of extract). All measurements were carried out in triplicate.

### 2.3. Instrumentation and Chromatographic Conditions

The phenolic composition of the ethanolic extract of UD was characterized using ultra-high-performance liquid chromatography–Orbitrap^®^ high-resolution mass spectrometry (UHPLC-Orbitrap^®^-HRMS) system [21]. Chromatographic analyses were conducted using a DIONEX UltiMate 3000 RS UHPLC system equipped with a pump, autosampler, and column oven, coupled to an Exactive Plus™ Orbitrap mass spectrometer (Thermo Fisher Scientific, Waltham, MA, USA) equipped with a heated electrospray ionization (HESI-II) probe. Prior to sample analysis, the mass spectrometer was calibrated with Pierce™ LTQ Velos ESI Positive Ion Calibration Solution and Pierce™ ESI Negative Ion Calibration Solution using an automatic syringe injector.

Separation of the analytes was achieved on a reversed-phase Cosmosil C18-EB column (100 × 2.1 mm, 3 μm). Gradient elution was performed using 0.5% (*v*/*v*) glacial acetic acid in ultrapure water as mobile phase A and LC-MS-grade methanol (99.9% purity; Sigma-Aldrich, St. Louis, MO, USA) as mobile phase B. Data acquisition was carried out in both positive and negative ionization modes. The Orbitrap-MS was operated under the following conditions: sheath gas flow rate, 35; auxiliary gas flow rate, 7; spray voltage, 3.5 kV; auxiliary gas and capillary temperature, 350 °C; S-lens RF level, 50; scan range, 60–800 *m*/*z*; resolution, 17,500; automatic gain control (AGC) target, 3 × 10^6^; maximum injection time, 2 ms; and collision energy, 25 eV.

### 2.4. Animals and Experimental Treatments

In this study, forty-eight healthy adult male Wistar Albino rats (2 months old, weighing 180–200 g) were used. Rats were divided into six groups with eight animals in each. Rats were used in the investigation in accordance with the permission of Local Ethics Committee of Animal Experiments in Hatay Mustafa Kemal University (Approval Number: 2024/02-03). The animals were kept under standard laboratory conditions (12 h light-12 h dark and 24 °C ± 3 °C). The rats were provided with tap water and chow ad libitum.

Control group: Rats received normal saline via oral gavage once daily for 56 consecutive days.

BPA group: BPA (CAS-No: 80-05-7, Sigma-Aldrich, St. Louis, MO, USA) was administered at a dose of 50 mg/kg by oral gavage once daily for 56 consecutive days [22].

UD100 group: Rats received UD at a dose of 100 mg/kg by oral gavage once daily for 56 consecutive days [23].

UD200 group: Rats received UD at a dose of 200 mg/kg by oral gavage once daily for 56 consecutive days [24].

BPA + UD100 group: Rats received 50 mg/kg BPA plus 100 mg/kg UD once daily orally for 56 consecutive days.

BPA + UD200 group: Rats received 50 mg/kg BPA plus 200 mg/kg UD once daily orally for 56 consecutive days. UD was administered 1 h before BPA administration [25]. Since the spermatogenic cycle in rats is completed in 52 days, the duration of administration was set at 56 days for all groups in this study [26].

Twenty-four hours after the last administration, the rats were weighed and deeply anesthetized with a xylazine (10 mg/kg, Rompun 2%, Bayer, Leverkusen, Germany)–ketamine (100 mg/kg, Keta-Control, Doğa İlaç, Istanbul, Türkiye) combination. The depth of anesthesia was confirmed by the absence of pedal withdrawal (toe pinch) and cutaneous pinch reflexes, as well as by the loss of jaw and skeletal muscle tone. Blood samples were then collected by cardiac puncture and centrifuged at 4000 rpm for 15 min at 4 °C to obtain serum, which was stored at −20 °C until biochemical analysis. Heart rate and respiratory rate were continuously monitored throughout the procedure. Euthanasia was subsequently completed by decapitation while the animals remained under deep anesthesia, in accordance with the approved animal ethics protocol. The reproductive organs (testes, epididymides, and seminal vesicles) were carefully dissected, cleared of adhering connective tissue, weighed using a digital balance, and assayed immediately. One testis was fixed in 10% neutral-buffered formalin for histopathological and immunohistochemical analyses, whereas the other was immediately frozen in liquid nitrogen and stored at −80 °C until molecular analyses.

### 2.5. Spermatological Analyses

Spermatological analyses (total sperm motility, sperm concentration, viability, plasma membrane integrity, and abnormal spermatozoa) were performed according to the methods described by Türk et al. [27] and Ezzatabadipour et al. [28], following the World Health Organization (WHO) [29] recommendations, with minor modifications. Both testicles were immediately cleared of surrounding tissues, and the epididymides were removed. The right and left cauda epididymides were minced separately in individual Petri dishes, each containing 500 µL of pre-warmed (37 °C) human tubal fluid (HTF) to allow spermatozoa to disperse into the medium. Sperm concentration was determined from the right cauda epididymis using a Thoma haemocytometer under a light microscope after 1:200 dilution of the sperm suspension, and spermatozoa were counted according to the procedure described by Türk et al. [27]. Other sperm quality parameters were assessed using the left cauda epididymis [27]. Total sperm motility was evaluated using a Makler counting chamber under a phase-contrast microscope equipped with a heated stage maintained at 37 °C (Olympus BX-51, Tokyo, Japan) at ×400 magnification. Motility was assessed in three microscopic fields, and the mean value was recorded as the final motility score [27,29]. For the assessment of sperm viability and morphology, 10 µL of sperm suspension was mixed with 10 µL of eosin–nigrosin stain (1.67 g eosin, 10 g nigrosin, 2.9 g sodium citrate, and 100 mL distilled water). Thin smears were prepared after approximately 1 min, air-dried, and examined under a light microscope at ×400 magnification. Live spermatozoa remained unstained, whereas dead spermatozoa were stained red. A total of 300 spermatozoa were evaluated per animal. Spermatozoa with abnormalities of the head, midpiece, or tail were classified as morphologically abnormal, and the percentage of abnormal spermatozoa was calculated [27,28,29]. The functional integrity of the sperm plasma membrane was evaluated using the hypo-osmotic swelling (HOS) test. Briefly, 30 µL of sperm suspension was added to 1 mL of hypo-osmotic solution containing 4.9 g sodium citrate and 9 g fructose in distilled water. The suspension was gently mixed and incubated at 37 °C for 60 min. Subsequently, 200 spermatozoa were examined under an inverted phase-contrast microscope at ×400 magnification. Spermatozoa exhibiting curled or swollen tails were considered HOS-positive, and the percentage of HOS-reactive spermatozoa was calculated [28].

### 2.6. Histopathological Analysis

Formalin-fixed testes were routine tissue processed (passing through an alcohol and xylene series for dehydration and fixation) and were then embedded in paraffin (Leica Biosystems, Wetzlar, Germany). Paraffin blocks were sectioned at a thickness of 4–5 μm on a rotary microtome (Leica RM2245, Leica Biosystems, Heidelberg, Germany), deparaffinized in xylene (Merck, Darmstadt, Germany), rehydrated through decreasing concentrations of alcohol, and stained with hematoxylin and eosin (H&E). Morphological changes were examined under a light microscope (Zeiss Imager A2, Oberkochen, Germany) and were then obtained microphotographs. Histopathological alterations were evaluated semi-quantitatively by a blinded histologist. Seminiferous tubular degeneration, germ cell loss, vacuolization, pyknotic nuclei, and interstitial fibrosis/edema were independently scored using a four-point scale: 0 = absent, 1 = mild (<25% of the examined area), 2 = moderate (25–50%), and 3 = severe (>50%). The total histopathological injury score was calculated as the sum of individual lesion scores (maximum score: 15). Five randomly selected non-overlapping fields per section were evaluated at ×200 magnification, and the average score for each animal was used for statistical analysis.

### 2.7. Immunohistochemical Analysis

Paraffin-embedded testicular tissue sections (5 µm thick) were incubated at 60 °C (Nüve EN 500, Istanbul, Türkiye) for 6 h. The sections were subsequently deparaffinized in xylene, rehydrated through a graded ethanol series, rinsed in distilled water, and washed with phosphate-buffered saline (PBS) (Sigma-Aldrich, St. Louis, MO, USA). Antigen retrieval process was performed by heating the sections in ethylenediaminetetraacetic acid (EDTA) solution (pH 8.0; Abcam, Waltham, MA, USA) using a microwave oven for three cycles of 6 min each. After PBS washing, endogenous peroxidase activity was quenched with 3% hydrogen peroxide (H_2_O_2_) solution (Thermo Fisher Scientific, Waltham, MA, USA), followed by blocking of non-specific binding sites with Ultra V Block solution (Thermo Fisher Scientific, Waltham, MA, USA). The sections were then incubated overnight at 4 °C with the following primary antibodies: Bcl-2-associated X protein (Bax; sc-20067), B-cell lymphoma 2 (Bcl-2; sc-7382), tumor necrosis factor-α (TNF-α; sc-52746), interleukin-1β (IL-1β; sc-12742), interleukin-6 (IL-6; sc-28343) (Santa Cruz Biotechnology, Santa Cruz, CA, USA), and cleaved caspase-3 (AF7022; Affinity Biosciences, Cincinnati, OH, USA). After the PBS washing step, the sections were incubated with the secondary antibody for 14 min. The sections were washed with PBS, incubated with streptavidin-peroxidase (Thermo Fisher Scientific, Waltham, MA, USA) for 15 min, and then incubated with 3,3′-diaminobenzidine (DAB) (Sigma-Aldrich, St. Louis, MO, USA) for approximately 7 min to visualize the immunoreaction. The sections were washed again with PBS and counterstained with Harris hematoxylin for approximately 1 min. Following PBS washing, the sections were dehydrated through a graded ethanol series, cleared in xylene. The section was covered with entellan (Merck, Darmstadt, Germany) before visualization and then imaged on Zeiss Imager A2 photomicroscope.

### 2.8. Western Blot Analysis

Frozen testis tissues were pulverized in liquid nitrogen and homogenized in radioimmunoprecipitation assay (RIPA) buffer supplemented with a protease/phosphatase inhibitor cocktail and nuclease (Thermo Fisher Scientific, Waltham, MA, USA). Following homogenization, the samples were centrifuged at 10,000× *g* for 30 min at 4 °C, and the supernatant was collected. Total protein quantities were determined using the bicinchoninic acid (BCA) protein assay kit (Thermo Fisher Scientific, Waltham, MA, USA). Equal amounts of protein (20 μg) were loaded onto a 10% TGX Stain-Free FastCast acrylamide gel (Bio-Rad, Hercules, CA, USA), separated by electrophoresis, and subsequently transferred onto polyvinylidene fluoride (PVDF) membranes (Bio-Rad, Hercules, CA, USA). After blocking with 5% skim milk (BioShop Canada Inc., Burlington, ON, Canada), the membranes were incubated overnight at 4 °C with the primary antibodies, followed by incubation with the secondary antibody for 1 h at room temperature. The membrane was treated with enhanced chemiluminescence (ECL) (Abbkine, Scientific Co., Ltd., Wuhan, China) and visualized with a ChemiDoc MP imaging system (Bio-Rad, Hercules, CA, USA). Band intensities were quantified using Image Lab software (version 6.1, Bio-Rad, Hercules, CA, USA), and protein expression levels were normalized to the β-actin, which served as the internal loading control (β-actin, sc-47778, Santa Cruz Biotechnology, Santa Cruz, CA, USA).

### 2.9. ELISA Assays for Oxidative Stress Markers and Testosterone in Rat Serum Samples

Serum catalase (CAT) and glutathione peroxidase (GPx) activities were determined using commercial colorimetric assay kits (Cat. No. E-BC-K031-M and E-BC-K096-M, respectively; Elabscience, Houston, TX, USA) according to the manufacturer’s instructions. All reactions were performed at 37 °C, and absorbance was measured at 405 and 412 nm for CAT and GPx, respectively, using a microplate reader (Multiskan Go, Thermo Fisher Scientific, Waltham, MA, USA). Serum superoxide dismutase (SOD) and testosterone levels were determined using commercial ELISA kits (Cat. No. ER1950 and ER1462, respectively; Fine Test, Wuhan, China) according to the manufacturer’s instructions. Assays were performed at 37 °C, and absorbance was measured at 450 nm using the microplate reader. Standard curves were generated using serial dilutions of the supplied standards (0, 0.781, 1.563, 3.125, 6.25, 12.5, 25, and 50 ng/mL for SOD and 0, 0.312, 0.625, 1.25, 2.5, 5, 10, and 20 ng/mL for testosterone).

### 2.10. Method Validation

Qualitative and quantitative analyses of phenolic constituents in the plant ethanolic extract were performed using a previously validated UHPLC-Orbitrap^®^-HRMS method under optimized analytical conditions [21]. Phenolic compounds were identified and quantified by external calibration using authentic reference standards over a concentration range of 10–100 ng/mL. The analytical method exhibited excellent linearity (R^2^ = 0.9903–0.9993), with limits of detection (LOD) ranging from 0.45 to 3.30 ng/mL and limits of quantification (LOQ) ranging from 1.50 to 11.00 ng/mL. Method accuracy was confirmed by recovery values ranging from 71.73% to 116.91%, demonstrating the reliability of the analytical procedure for phytochemical profiling and quantification.

### 2.11. Statistical Analysis

Statistical analysis of spermatological parameters was performed using SPSS 23 program (SPSS Inc.; Chicago, IL, USA). All other statistical analyses were performed using GraphPad Prism (version 10, San Diego, CA, USA). Data are presented as mean ± standard deviation (SD). Data with normal distribution among multiple groups were analyzed using One-Way ANOVA, and post hoc analysis was conducted using Tukey HSD test. One-Way ANOVA and Duncan’s test were used to evaluate the statistical differences in spermatological parameters between groups. Histopathological scores were expressed as median (minimum–maximum). Differences among groups were analyzed using the Kruskal–Wallis test followed by Dunn-Bonferroni post hoc multiple comparisons. A *p*-value less than 0.05 was considered statistically significant.

## 3. Results

### 3.1. Determination of TPC in the UD Extract

The TPC of the UD ethanolic extract was determined to be 6.77 ± 0.005 mg GAE/g.

### 3.2. Reproductive Organ Weights and Gonadosomatic Index (GSI) Evaluation

The weights of testes, epididymis, and seminal vesicles, and GSI results are presented in Table 1. When the right and left testes were weighed, the highest values were observed in the BPA and BPA + UD100 groups, while the lowest values were found in the control group (*p* < 0.05). When the right epididymis was weighed, the lowest values were observed in the UD100 and UD200 groups, and when the left epididymis was weighed, the lowest values were found in the UD100 and BPA + UD100 groups (*p* < 0.05). The weight of seminal vesicles was found to be significantly lower in the UD100 group compared to the other experimental groups (*p* < 0.05). The GSI was observed to be significantly lower in the BPA group compared to the other experimental groups (*p* < 0.05).

### 3.3. Evaluation of Effect of BPA and UD Extract Administration on Sperm Parameters

Spermatological analysis results are shown in Table 2. No statistically significant difference in sperm concentration was observed between the groups. The lowest values for motility and membrane integrity were observed in the BPA group (*p* < 0.05). The highest values in the assessment of abnormal and dead spermatozoa ratio were detected in the BPA group (*p* < 0.05). It was determined that BPA administration in rats negatively affected concentration, motility, membrane integrity and increased the rates of abnormal and dead spermatozoa. In groups treated with UD at doses of 100 mg/kg and 200 mg/kg following BPA-induced damage, improvements in concentration, motility, and membrane integrity were observed. Additionally, it was determined that the increased proportion of abnormal and dead spermatozoa resulting from BPA administration was reduced in the groups treated with UD at doses of 100 mg/kg and 200 mg/kg.

### 3.4. Analysis of Phenolic Compounds of UD Ethanolic Extract with UHPLC-Orbitrap^®^-HRMS

Table 3 shows the quantitative analysis and profiling of the phenolic compounds identified using UHPLC-Orbitrap^®^-HRMS in the ethanolic extract of UD. The study investigated the presence of phenolic compounds in the UD extract using 87 phenolic reference standards. Following qualitative and quantitative analyses, 11 of these phenolic compounds were identified in the UD extract. The two most abundant components identified in the UD extract were quinic acid (6807.55 µg/g of extract) and chlorogenic acid (1216.356 µg/g of extract). Protocatechuic acid, chrysin, vicenin-2, schaftoside, luteolin-7-rutinoside, quercetin, vitamin C, esculin hydrate and rosmarinic acid were among the other phenolic compounds identified in the ethanolic extract of UD. Moreover, Table 3 lists the concentration ranges (µg/g of extract) of the phenolic compounds in the extract, as well as their retention times (RT), Quan peak (*m*/*z*) values, and curve types.

### 3.5. Histopathological Findings

**Figure 1:**
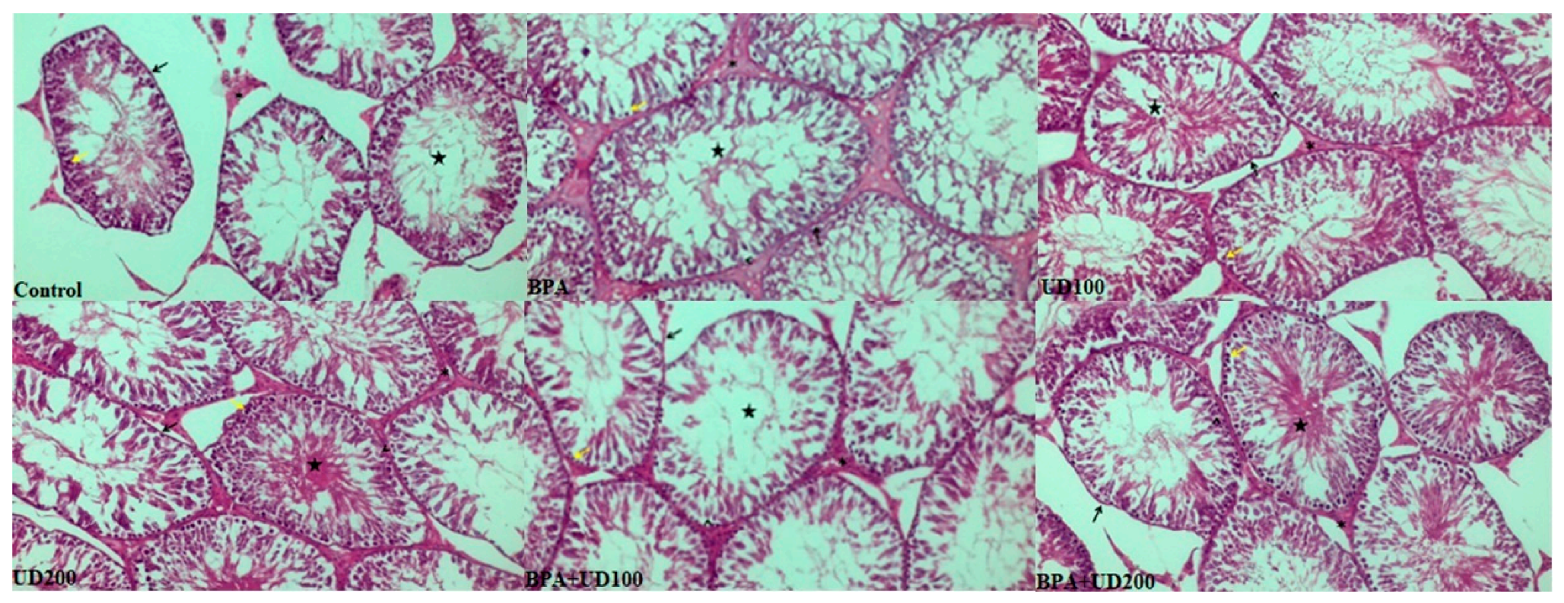
Histopathological staining of testicular sections. Black arrow: seminiferous tubule basal membrane, yellow arrow: spermatogonia, arrowhead: Sertoli cell, *: interstitial area, star: seminiferous tubule lumen. (Scale bar: 50 µm, Hematoxylin-Eosin staining, magnification: 20×).

Histopathological findings of the testicular tissues of rats in the control and experimental groups are presented in Figure 1. In the testicular sections of the control, UD100, and UD200 groups, the seminiferous tubules were observed to be round or oval in shape with distinct boundaries, and the stages of spermatogenic cells were found to be organized and at different developmental stages. Sertoli cells within the tubules and Leydig cells in the interstitial areas were prominently visible. In the UD200 group sections, the presence of spermatozoa in the seminiferous tubule lumen was notably evident. No pathology was observed in the interstitial area. In the sections of the BPA group, severe testicular pathology was noted. The seminiferous tubules exhibited degenerative alterations and loss of structural integrity. Degeneration and pyknotic nuclei were present in the spermatogenic cells. A reduction in the diameter of the seminiferous tubules and a decrease in the number of germ cells were observed. Increased prominence of the intertubular connective tissue, along with the presence of vacuoles and fibrosis, was noted. In the testicular sections of the BPA + UD100 group, an improvement in testicular histology was observed compared to the BPA group. Structural integrity of the seminiferous tubule membrane was restored, and the spermatogenic cell line was observed to extend from the basal membrane toward the lumen. It was observed that fibrosis and vacuoles in the interstitial area decreased. Following BPA administration, UD200 treatment was observed to provide the most effective therapy compared to the BPA and BPA + UD100 groups. The seminiferous tubules and spermatogenic cells exhibited a histological structure similar to the control group. The presence of Leydig cells was noted in the interstitial area. A high density of spermatozoa was observed in the seminiferous tubule lumen.

Semi-quantitative histopathological scoring supported the qualitative microscopic observations (Table 4). The total histopathological injury score differed significantly among the experimental groups (*p* < 0.001). The BPA group exhibited the highest histopathological injury score, indicating severe seminiferous tubular degeneration, germ cell loss, vacuolization, pyknotic nuclei, and interstitial fibrosis/edema. In contrast, both BPA + UD100 and BPA + UD200 treatments significantly reduced testicular injury compared with the BPA group, with the BPA + UD200 group demonstrating the greatest histological improvement. No significant differences were observed among the control, UD100, UD200, and BPA + UD200 groups, indicating that the higher dose of UD restored the histological architecture to a level comparable with that of normal testicular tissue.

### 3.6. Immunohistochemical Results

**Figure 2:**
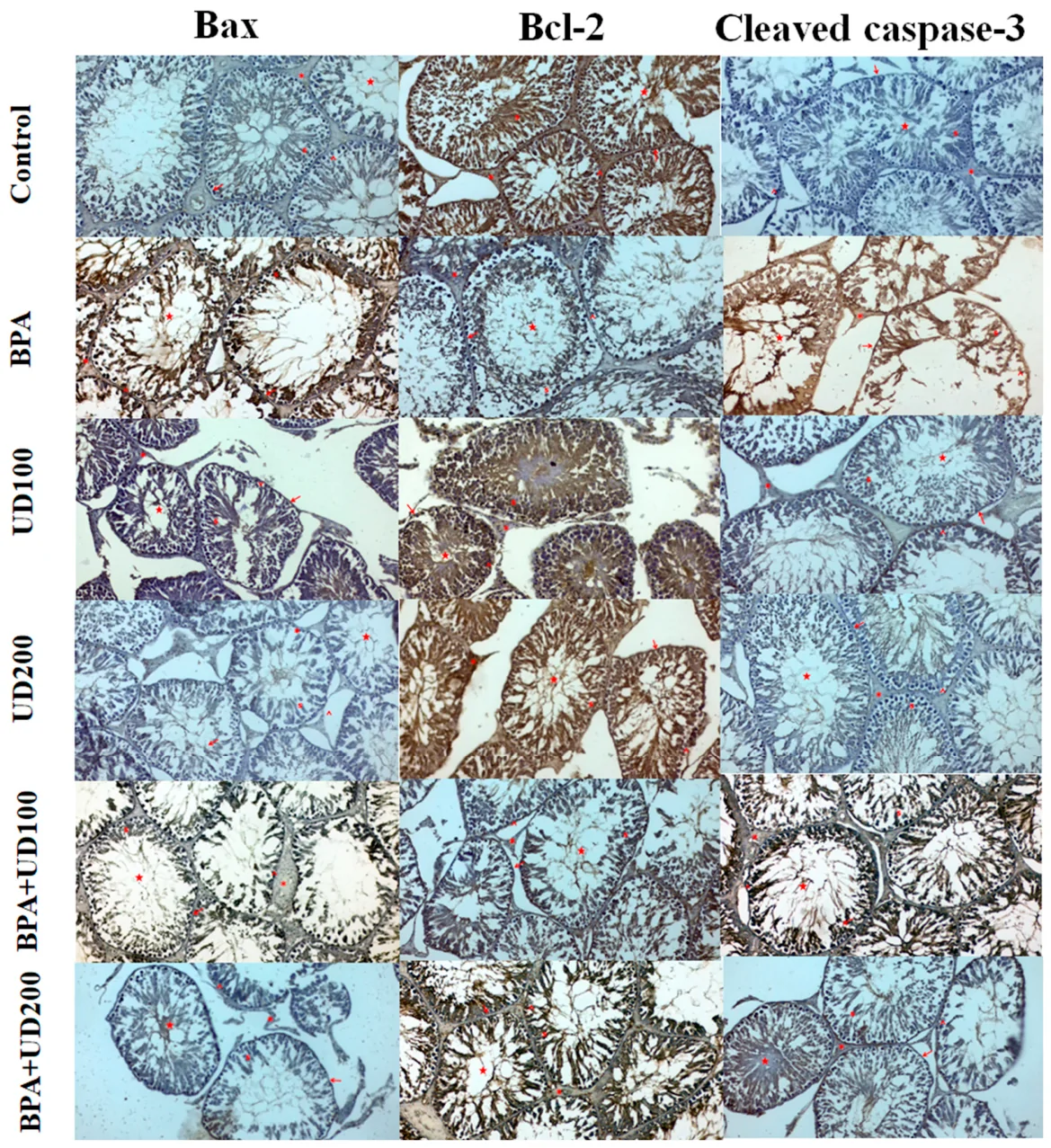
The Bax, Bcl-2, and cleaved caspase-3 immunohistochemical staining of testicular sections in the control and experimental groups. Arrow: seminiferous tubule basal membrane, arrowhead: Sertoli cell, s: spermatogenic cells, *: interstitial space, star: seminiferous tubule lumen. (Bar: 50 µm, Bax, Bcl-2, cleaved caspase-3 immunostaining, magnification: 20×). Bax, Bcl-2-associated X protein; Bcl-2, B-cell lymphoma 2; BPA, bisphenol A; UD, *Urtica dioica*.

Figure 2 shows the Bax, Bcl-2, and cleaved caspase-3 immunostaining of testicular tissues in the control and experimental groups. In the testicular tissue sections of the control, UD100, and UD200 groups, negative Bax and cleaved caspase-3 expressions were noted in the seminiferous tubule basal membrane, spermatogenic and Sertoli cells, and Leydig cells in the interstitial area, while strong positive Bcl-2 expression was observed. BPA administration was found to upregulate Bax and cleaved caspase-3 expressions compared to the control, UD100, and UD200 groups, while downregulating Bcl-2 expression was noted. Additionally, positive Bax and cleaved caspase-3 expressions were observed in the seminiferous tubule basal membrane, spermatogenic and Sertoli cells, and interstitial Leydig cells, while negative Bcl-2 expression was noted. Following BPA administration, UD100 and UD200 treatments were observed to decrease Bax and cleaved caspase-3 expression while increasing Bcl-2 expression in the seminiferous tubule basal membrane, interstitial Leydig cells, spermatogenic cells, and Sertoli cells.

**Figure 3:**
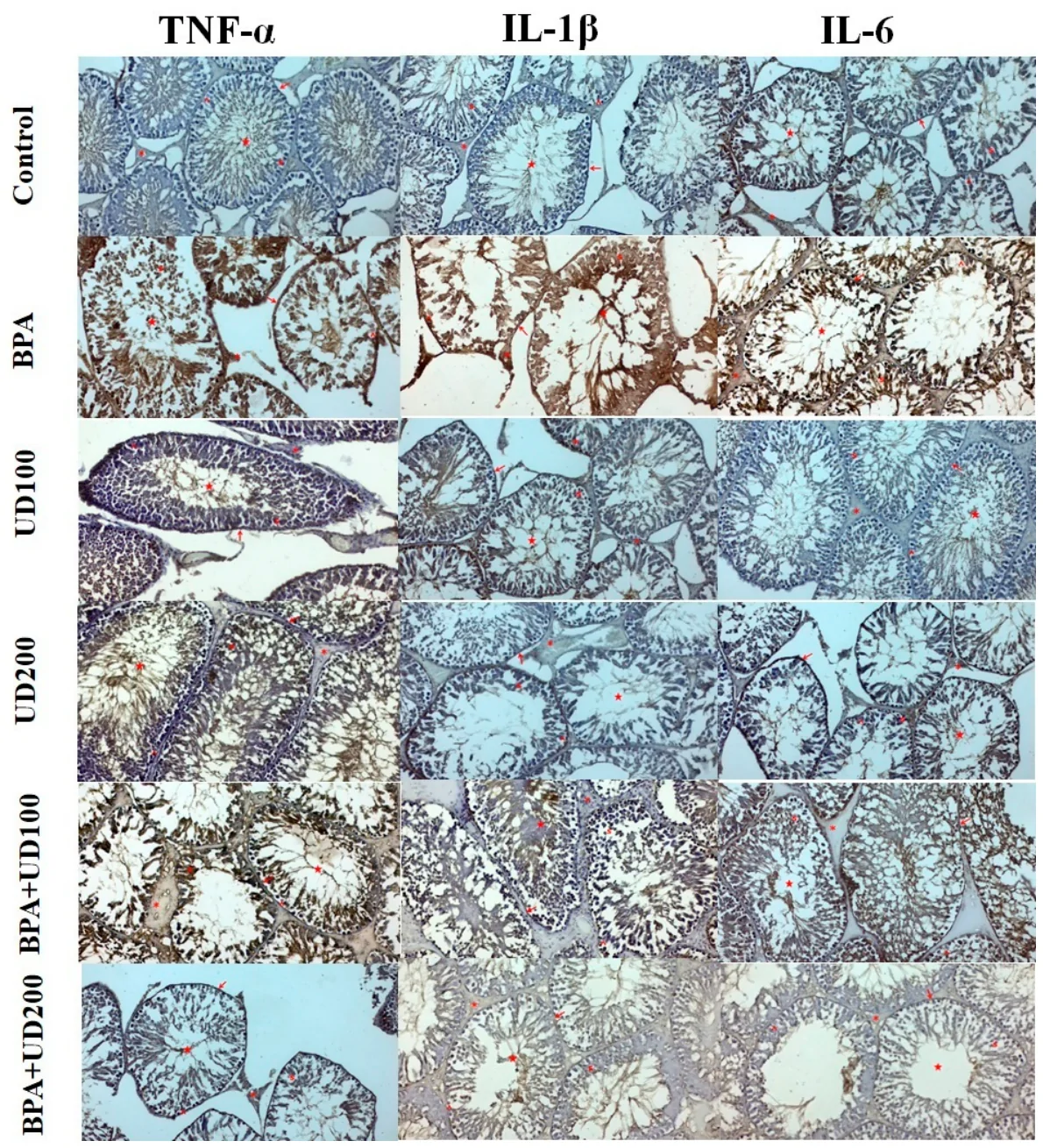
The TNF-α, IL-1β, and IL-6 immunohistochemical staining of testicular sections in the control and experimental groups. Arrow: seminiferous tubule basal membrane, arrowhead: Sertoli cell, s: spermatogenic cells, *: interstitial space, star: seminiferous tubule lumen. (Bar: 50 µm, TNF-α, IL-1β, and IL-6 immunostaining, magnification: 20×). TNF-α, tumor necrosis factor alpha; IL-1β, interleukin-1 beta; IL-6, interleukin-6; BPA, bisphenol A; UD, *Urtica dioica*.

Figure 3 shows the TNF-α, IL-1β, and IL-6 immunostaining of testicular tissues in the control and experimental groups. In the testicular tissue sections of the control, UD100, and UD200 groups, negative immunoreactivity for TNF-α, IL-1β, and IL-6 was observed in the seminiferous tubule basal membrane, spermatogenic and Sertoli cells, and Leydig cells in the interstitial area. BPA administration was observed to upregulate the expression of TNF-α, IL-1β, and IL-6 compared to the control, UD100, and UD200 groups. Positive TNF-α, IL-1β, and IL-6 expressions were observed in the seminiferous tubule basal membrane, spermatogenic and Sertoli cells, and interstitial Leydig cells. Following BPA administration, UD100 and UD200 treatments were observed to downregulate the expression of TNF-α, IL-1β, and IL-6. In the seminiferous tubule basal membrane, spermatogenic and Sertoli cells, and interstitial Leydig cells, the expression of TNF-α, IL-1β, and IL-6 was observed to be significantly reduced. However, following BPA exposure, UD200 treatment was found to be more effective than UD100 treatment and significantly downregulated the expression of TNF-α, IL-1β, and IL-6.

### 3.7. Western Blotting Analysis Results

**Figure 4:**
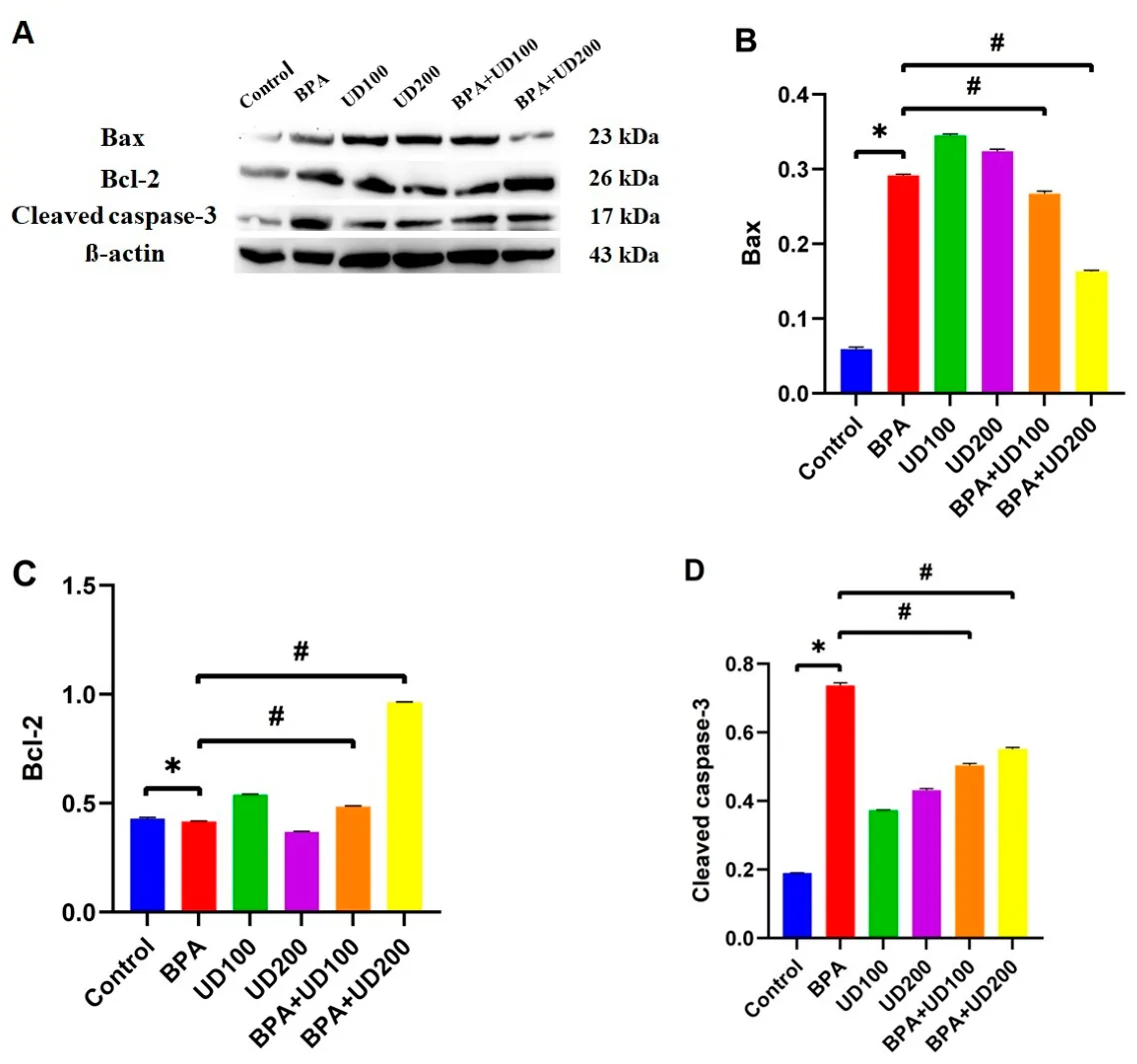
Bax, Bcl-2, and cleaved caspase-3 protein expression levels in the testicular tissues from control and experimental groups. Quantification of protein band optical densities to determine Bax, Bcl-2, cleaved caspase-3, and β-actin levels. (**A**) Western blot bands of Bax, Bcl-2, cleaved caspase-3, and β-actin; (**B**) Bax protein expression; (**C**) Bcl-2 protein expression; (**D**) cleaved caspase-3 protein expression. Bars represent the mean ± SD of experiments (n = 8 per group). (* *p* < 0.05, compared to the control group) (# *p* < 0.05, compared to the BPA group). Bax, Bcl-2-associated X protein; Bcl-2, B-cell lymphoma 2; BPA, bisphenol A; UD, *Urtica dioica*. (See Supplementary Materials).

Expression levels of the Bax, Bcl-2, cleaved caspase-3, TNF-α, IL-1β, and IL-6 proteins in testicular tissue were determined using Western blot analysis. Figure 4 shows the expression levels of the Bax, Bcl-2, and cleaved caspase-3 proteins in testicular tissues. It was observed that the Bax and cleaved caspase-3 levels were significantly increased in the BPA group compared to the control group, while the Bcl-2 level was significantly decreased (*p* < 0.05). In the BPA + UD100 and BPA + UD200 groups, it was observed that the expression levels of Bax and cleaved caspase-3 proteins were significantly reduced compared to the BPA group, while the expression levels of Bcl-2 protein were significantly increased (*p* < 0.05) (Figure 4).

**Figure 5:**
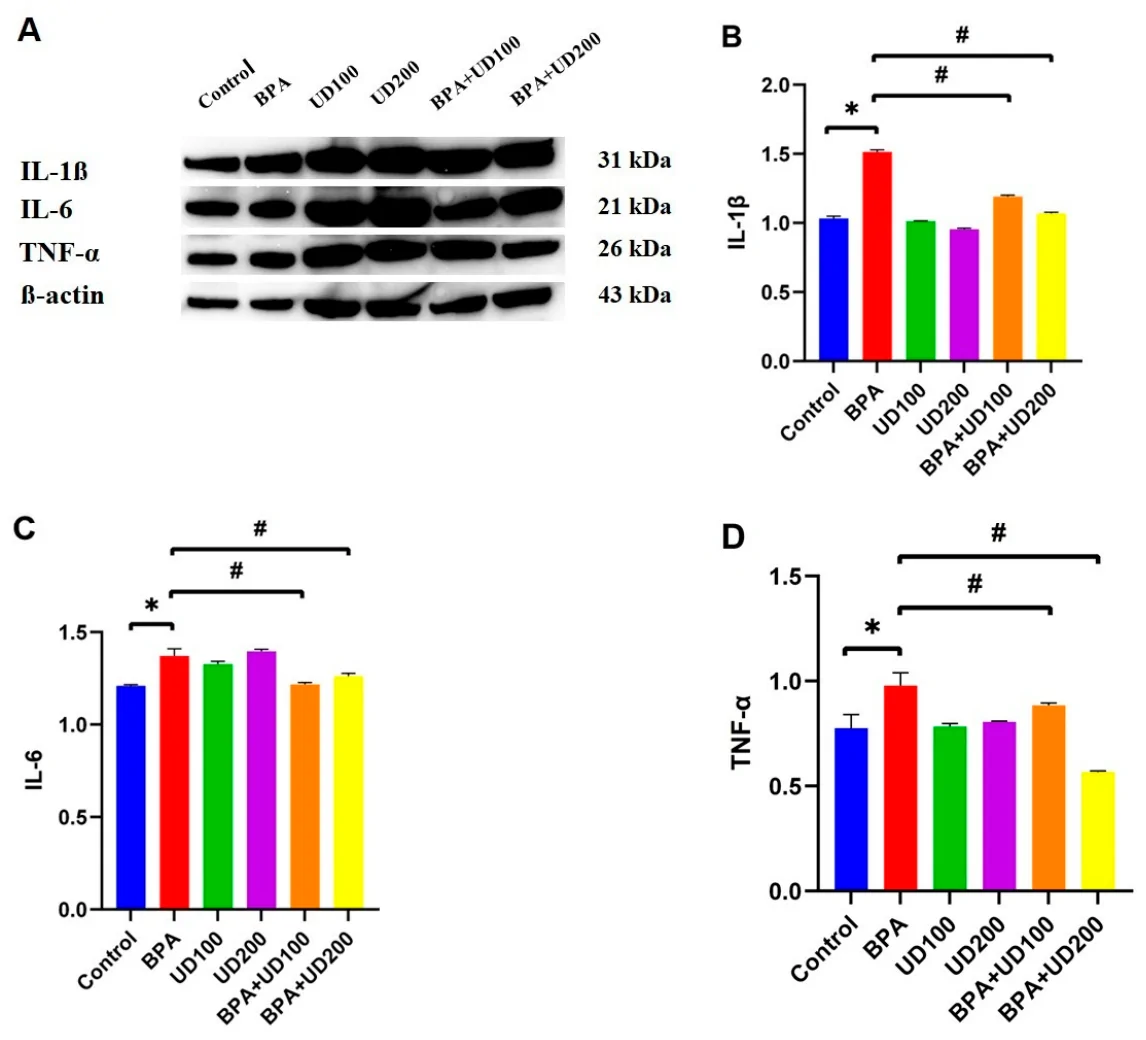
TNF-α, IL-1β, and IL-6 protein expression levels in the testicular tissues from control and experimental groups. Quantification of protein band optical density to determine TNF-α, IL-1β, IL-6, and β-actin levels. (**A**) Western blot bands of IL-1β, IL-6, TNF-α, and β-actin; (**B**) IL-1β protein expression; (**C**) IL-6 protein expression; (**D**) TNF-α protein expression. Bars represent the mean ± SD of experiments (n = 8 per group). (* *p* < 0.05, compared to the control group) (# *p* < 0.05, compared to the BPA group). TNF-α, tumor necrosis factor alpha; IL-1β, interleukin-1 beta; IL-6, interleukin-6; BPA, bisphenol A; UD, *Urtica dioica*. (See Supplementary Materials).

Figure 5 shows the expression levels of TNF-α, IL-1β, and IL-6 proteins in testicular tissues. It was observed that the TNF-α, IL-1β, and IL-6 levels were significantly increased in the BPA group compared to the control group (*p* < 0.05). In the BPA + UD100 and BPA + UD200 groups, it was observed that the expression levels of TNF-α, IL-1β, and IL-6 proteins were significantly reduced compared to the BPA group (*p* < 0.05) (Figure 5).

### 3.8. CAT, SOD, GPx, and Testosterone Levels in Rat Serum Samples

**Figure 6:**
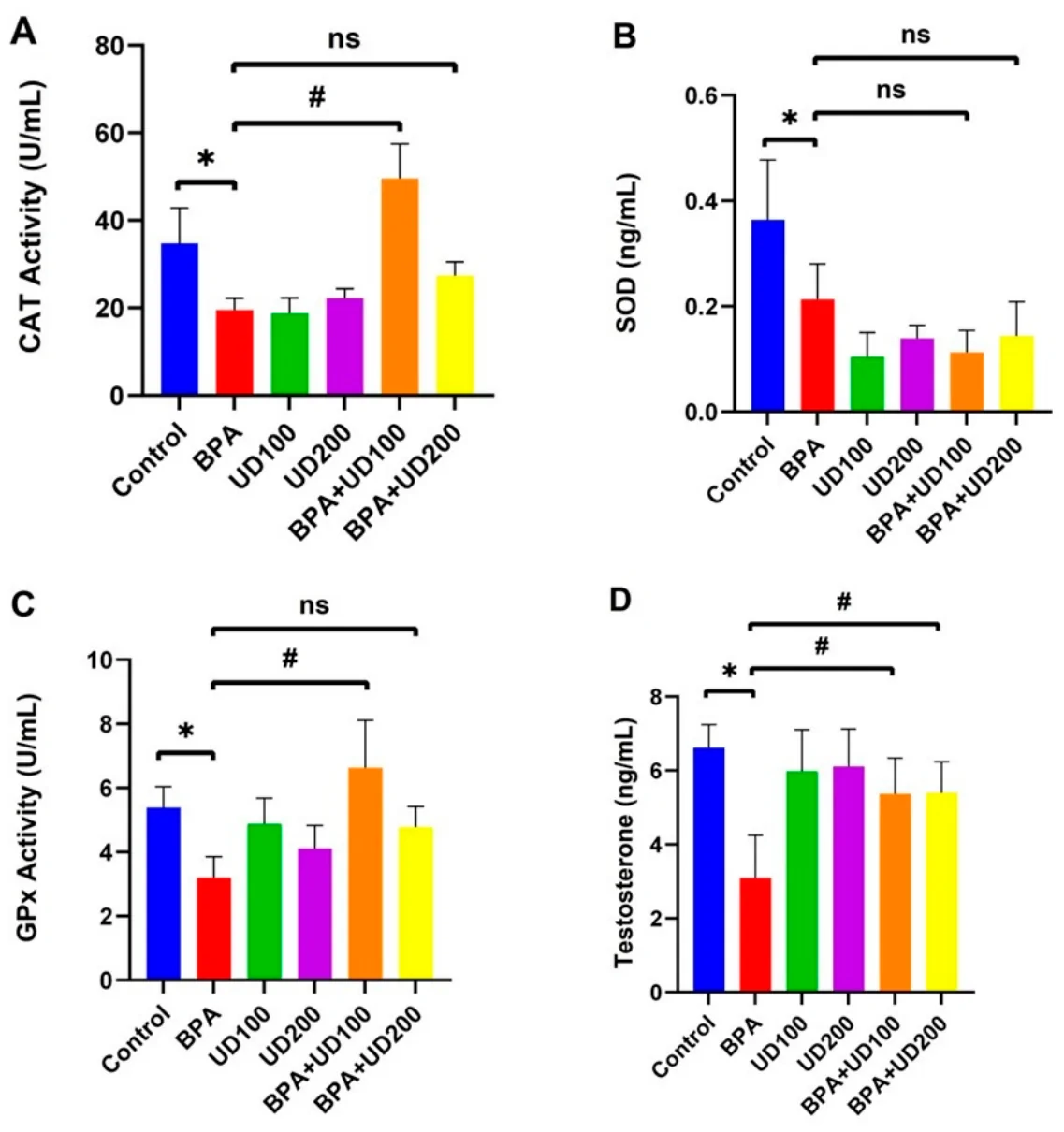
Serum (**A**) CAT, (**B**) SOD, (**C**) GPx, and (**D**) testosterone levels in control and experimental rat groups. Bars represent the mean ± SD of experiments (n = 8 per group). (* *p* < 0.001, compared to the control group) (# *p* < 0.001, compared to the BPA group). CAT, catalase; SOD, superoxide dismutase; GPx, glutathione peroxidase; ns, not significant; BPA, bisphenol A; UD, *Urtica dioica*.

CAT enzyme activity measured in rat serum samples is presented in Figure 6A. Enzyme activity is expressed as U/mL. The control group showed an average CAT activity of 34.78 ± 8.05 U/mL. Exposure to BPA significantly decreased CAT activity to 19.49 ± 2.74 U/mL (*p* < 0.001). However, co-administration of 100 mg/kg UD (BPA + UD100) significantly increased CAT activity to 49.55 ± 7.98 U/mL compared to the BPA group (*p* < 0.001). Similarly, co-administration with 200 mg/kg UD (BPA + UD200) raised CAT activity to 27.35 ± 3.12 U/mL, also higher than in the BPA group, though less effective than the 100 mg/kg dose.

SOD levels measured in rat serum samples are presented in Figure 6B. SOD levels are expressed as ng/mL. The control group exhibited the highest average SOD concentration (0.36 ± 0.11 ng/mL), whereas BPA exposure led to a significant reduction (0.21 ± 0.07 ng/mL) (*p* < 0.001). Treatment with UD100 and UD200 alone resulted in further reductions in SOD levels (0.10 ± 0.05 ng/mL and 0.14 ± 0.02 ng/mL, respectively). Combined administration of BPA with UD100 (0.11 ± 0.04 ng/mL) or UD200 (0.14 ± 0.06 ng/mL) did not lead to a significant recovery of SOD levels compared to BPA administration alone.

GPx levels measured in rat serum samples are presented in Figure 6C. GPx levels are expressed as U/mL. Comparative statistical analysis revealed significant difference between control (5.39 ± 0.65 U/mL) and BPA (3.19 ± 0.66 U/mL) groups (*p* < 0.001), suggesting that BPA exposure led to a significant decrease in GPx levels. Moreover, co-administration with UD100 (6.63 ± 1.48 U/mL) significantly increased GPx activity compared to the BPA group (*p* < 0.001). Although the co-administration with UD200 (4.78 ± 0.65 U/mL) increased GPx levels compared to the BPA group, no significant difference was found between the BPA and BPA + UD200 groups.

Testosterone concentrations in rat serum samples are presented in Figure 6D. Testosterone levels are expressed as ng/mL. Compared with the control group (6.61 ± 0.81 ng/mL), BPA (3.08 ± 1.16 ng/mL) significantly reduced testosterone levels (*p* < 0.001). In the groups treated with UD100 and UD200 alone, testosterone levels were similar to those in the control group, indicating that both doses were effective in maintaining normal testosterone levels (5.99 ± 1.13 ng/mL and 6.11 ± 1.05 ng/mL, respectively). Compared with the BPA group, the testosterone levels in the BPA + UD100 (5.37 ± 0.97 ng/mL) and BPA + UD200 (5.39 ± 0.89 ng/mL) groups were significantly higher (*p* < 0.001).

## 4. Discussion

Exposure to environmental contaminants in men may lead to infertility, one of the most significant problems affecting sociocultural life [30]. Exposure to BPA, which is the building block of polycarbonate compounds frequently used in daily life, causes a decrease in sperm count and testosterone levels by triggering oxidative stress [26,31]. Epidemiological studies have shown that exposure to BPA especially causes changes in hormonal levels, which significantly contributes to male infertility [32,33]. It is known that factors such as oxidative stress, inflammation, and apoptosis play a role in the pathophysiology of male infertility, which has a highly complex etiology [30]. Acısu et al. [34] reported that, consistent with our current study, BPA administration resulted in a decrease in testosterone level, sperm motility and concentration, and Bcl-2 level, while there was an increase in abnormal spermatozoa and Bax levels. In another study conducted by Malmir et al. [26], it was also indicated that BPA administration caused low sperm viability and count, as well as decreased testosterone levels. It was reported that BPA exposure reduced sperm production, sperm reserves and transit time in the epididymis, and testosterone concentration, and caused damage to the plasma membrane and acrosome integrity of sperm cells, and an increase in sperm defects [35].

Oxidative stress causes reproductive abnormalities by triggering the disruption of the normal apoptotic process. Apoptosis, known as programmed cell death, normally plays an important role in preventing developmental anomalies and various diseases; however, when uncontrollably activated by ROS, it can lead to infertility by causing impairments in sperm cells and sperm quality parameters [30,36]. In the normal spermatogenesis process, apoptosis is responsible for eliminating damaged germ cells, thereby controlling their uncontrolled proliferation [37]. Ryu et al. [8] demonstrated that BPA exposure, even at very low levels, caused an increase in Bax levels and a significant decrease in Bcl-2 and testosterone levels compared to the control group. Similarly, in a different study by Zhang et al. [38], it was demonstrated that BPA suppresses SOD and GPx antioxidant enzyme levels, leading to an increase in Bax levels and a decrease in Bcl-2 and testosterone levels. It is known that there is a significant relationship between reproductive physiology disorders and ROS production [13]. Oxidative stress, recognized as a cause of many diseases such as infertility, is defined as an imbalance between pro-oxidants and antioxidants. In order to maintain normal testicular activity, excessive ROS is continuously neutralized and kept under control [39]. However, when ROS levels during the spermatogenesis process exceed the levels of CAT, SOD, and GPx, which are key regulators of cellular defense, it leads to pathological conditions such as sperm dysfunction and reduced sperm count, DNA damage in sperm cells, and testicular structural disorders, thereby impairing the reproductive process [40,41]. Similarly to our results, Wang et al. [25] emphasized that BPA caused oxidative damage by reducing antioxidant enzyme levels such as SOD and GPx in testicular tissue, leading to disorganization in the seminiferous tubules and a decrease in the number of mature sperm and germ cells. Our findings show that oxidative damage and inflammation caused by BPA exposure led to a decrease in the activity of antioxidant enzymes, resulting in disorders such as degeneration in testicular structures, seminiferous tubules, and cell morphology, and that this condition may play a significant role in the development of infertility by causing a decrease in germ cell numbers by inducing apoptosis in cells, and consequently decreasing testosterone levels.

As demonstrated in recent studies, various agents have been used to prevent the toxic effects of BPA on male reproductive physiology; however, the effect of UD has not been previously investigated. One of the strengths of our study is that it demonstrates not only the effect of UD on the damage caused by BPA on testicular tissue, sperm, and hormonal parameters, but also its effect on proteins involved in ROS levels, apoptosis, and inflammation, which are among the triggering pathways of this damage. It has been emphasized that, in order to achieve success in the treatment of infertility, it may be more beneficial to develop therapeutic strategies targeting the pathological mechanisms involved in the formation of sperm damage [30]. There are numerous studies in the literature in which UD has been used against testicular damage induced by various toxic substances. Siouda and Abdennour [17] demonstrated that UD administration in rats with mercury-induced testicular damage supported an increase in sperm motility and concentration, as well as in the levels of GSH and testosterone. Additionally, they showed that deformations and degenerations in the testicular and epididymal tissues of rats disappeared, resulting in a restored normal and homogeneous appearance. Similarly to our results, Albogami [13] reported that treatment with UD following testicular damage increased GPx levels, thereby enhancing plasma testosterone levels, sperm count, and motility, reducing the percentage of abnormal sperm, and providing moderate histopathological improvement. A clinical study also emphasized that UD was effective in improving parameters such as sperm volume, morphology, and motility in infertile men [42]. It was reported that UD treatment in damaged testes improved sperm integrity, quality, quantity, and testosterone concentration [18,43]. In the present study, similar to previous studies, UD treatment was reported to have beneficial effects on histopathological changes in testicular tissue, including improved seminiferous tubule integrity, increased regularity and number of spermatogenic cells, and restoration of the epithelial thickness of the tubules [44].

One of the notable findings of our study is that while UD extract administered at a dose of 100 mg/kg following BPA-induced testicular damage resulted in a more pronounced increase in CAT and GPx activities, the extract administered at a dose of 200 mg/kg produced a more pronounced improvement in sperm parameters and histopathological lesions. This difference can be explained by the complex pharmacokinetic and pharmacodynamic properties of the polyphenols present in the UD extract, as well as the fact that different biological mechanisms of action become dominant at different dose levels and that some biological responses reach a saturation point [45]. Due to the pharmacokinetic properties of polyphenols, bioavailability does not always increase linearly with the administered dose, and absorption or systemic exposure of certain bioactive compounds may vary at specific doses [46,47]. Therefore, administering higher oral doses may not result in a simultaneous and proportional improvement in all experimental parameters. Furthermore, it has been reported that the protective effects of high doses of plant extracts are not limited solely to the activation of enzymes involved in the endogenous antioxidant defense system but may also occur through the simultaneous modulation of various signaling pathways, particularly inflammation and apoptosis. In this regard, it can be stated that the 100 mg/kg UD dose is more effective in stimulating CAT and GPx activities, whereas the 200 mg/kg UD dose exhibits a stronger protective effect against BPA-induced sperm and testicular tissue damage. It is thought that this protective effect may be related to more effective modulation of inflammatory and apoptotic pathways, as supported by the decrease in TNF-α, IL-1β, IL-6, Bax, and cleaved caspase-3 expression and the increase in Bcl-2 expression observed in our study.

Our findings revealed that BPA significantly reduced SOD levels, and treatment with UD100 or UD200 did not provide a statistically significant protective benefit under the tested conditions. The fact that no improvement in SOD level was observed in our study is consistent with previous reports indicating that, despite the administration of various protective agents in experimental models of testicular toxicity, no significant improvement in SOD level was observed. In support of our findings, it has been reported that no significant improvement in SOD level was observed among treated groups in experimental models of lead-induced testicular damage [48,49]. Similarly, in vitro studies conducted on germ cells have shown that phytochemical treatments do not modulate SOD level and do not cause a significant change in this parameter [50]. These findings suggest that the antioxidant activity of UD may not regulate all antioxidant enzymes equally and that oxidative balance may be restored through different antioxidant defense mechanisms.

Cytokines, which are natural components of seminal plasma, play a significant role in maintaining normal physiological functions such as spermatogenesis and hormonal regulation in the male reproductive system [41,51]. It has been emphasized that data on the impact of cytokines, which are involved in intercellular communication, on male infertility are limited [39]. The increased production of cytokines, particularly TNF-α, IL-1β, and IL-6, in seminal plasma following inflammation in testicular tissue triggers excessive ROS production and enhances spermatozoa membrane peroxidation, thereby negatively affecting fertility [52]. Additionally, clinical studies have reported that high levels of proinflammatory cytokines such as TNF-α are associated with low serum testosterone concentrations [53]. The presence of high levels of specific cytokines in seminal plasma is frequently associated with a reduction in sperm quality [54]. Studies support that cytokines, which act as mediators of oxidative damage, can affect sperm quality and thus male fertility [52]. Another study demonstrated that BPA-induced damage increased the levels of TNF-α, IL-1β, and IL-6, which in turn enhanced ROS production, leading to sperm damage, consistent with our findings [55]. Similarly, Çibuk et al. [56] emphasized that BPA administration caused an increase in IL-1β, IL-6, and TNF-α cytokines, reducing antioxidant capacity and resulting in oxidative damage. Cytokines induce redox imbalance in sperm cells, indirectly leading to apoptosis [41]. Consistent with our study results, it was reported that the cytokines such as TNF-α, IL-1β, and IL-6 induced in testicular tissue following BPA administration suppressed CAT, SOD, and GPx, leading to a decrease in Bcl-2 levels and an increase in Bax and caspase-3 protein expression levels, thereby triggering apoptosis. This was noted to cause a reduction in sperm concentration, motility, and testosterone levels, as well as an increase in the number of abnormal and dead sperm [57].

On the other hand, this study has several limitations. First, the protective effect of UD against BPA-induced reproductive toxicity was investigated only in a male rat model. Therefore, the findings cannot be directly extrapolated to other male animal species, such as rams, bulls, and bucks. Accordingly, further experimental studies involving different animal species are needed to determine whether the protective effects of UD are consistent across species and to enhance the translational value of the present findings. Another limitation is that although BPA-induced testicular damage including oxidative stress, inflammation, and apoptosis was comprehensively evaluated using biochemical, molecular, immunohistochemical, and histopathological analyses, the hierarchical molecular relationship among these processes could not be directly established. It has not been experimentally demonstrated at the molecular level how BPA-induced excessive ROS production modulates upstream signaling pathways such as NF-κB, Nrf2, and HO-1 that play a role in regulating oxidative stress and inflammatory responses, and how these pathways direct the signaling cascade leading to testicular structural abnormalities, abnormal sperm, and reduced testosterone secretion has not been experimentally demonstrated at the molecular level. Furthermore, although quinic acid and chlorogenic acid have been identified as the predominant phenolic compounds in the UD ethanolic extract, the individual contributions of these compounds to antioxidant, anti-inflammatory, and antiapoptotic effects have not been evaluated. However, it would be more appropriate to consider UD’s protective efficacy against BPA-induced testicular damage as a result of the synergistic and collective effects of the bioactive compounds contained in the extract, rather than the effect of a specific bioactive compound. This study was conducted using an in vivo experimental testis damage model; direct verification of the molecular targets of UD polyphenols at the cellular level was beyond the scope of this study. Therefore, supporting future in vitro studies with pathway-specific molecular analyses will contribute to a more comprehensive elucidation of the direct cellular targets responsible for UD’s protective effects and the underlying signaling mechanisms. Additionally, the study did not evaluate the high-dose administration of UD through chronic toxicity tests, nor did it elucidate the intestinal absorption, bioavailability, and pharmacokinetic properties of its phenolic components. Therefore, further pharmacokinetic and chronic toxicity studies are necessary to facilitate the clinical application of UD as a potential therapeutic agent against BPA-induced testicular toxicity. Additionally, the present study did not establish a direct component–efficacy relationship between the individual phytochemical constituents identified in the UD extract and the observed biological effects. Since the phytochemical analysis was conducted to characterize the constituent profile of the UD extract, correlations between individual phenolic compounds and pharmacological outcomes could not be assessed.

## 5. Conclusions

In conclusion, our findings demonstrate that UD treatment suppresses oxidative stress, inflammation, and apoptosis induced in testicular tissues due to BPA exposure. This study provides evidence that UD, with its antioxidant activity, may exert a therapeutic effect in the treatment of male infertility caused by BPA-induced testicular dysfunction. Further clinical studies are needed to establish UD’s potential use as a pharmacological agent against BPA-induced toxicity.

## Figures and Tables

**Table 1:** Effect of BPA and UD on testes, epididymis, and seminal vesicles weight (g), and GSI.

Groups	Right Testicles	Left Testicles	Right Epididymis	Left Epididymis	Seminal Vesicles	GSI (%)
Control	1.70 ± 0.03 ^c^	1.72 ± 0.02 ^d^	0.36 ± 0.01 ^a^	0.38 ± 0.02 ^a^	1.26 ± 0.04 ^d^	0.47 ± 0.03 ^a^
BPA	1.84 ± 0.05 ^a^	1.88 ± 0.03 ^a^	0.38 ± 0.01 ^a^	0.37 ± 0.02 ^a^	1.69 ± 0.02 ^a^	0.44 ± 0.03 ^c^
UD100	1.81 ± 0.05 ^b^	1.83 ± 0.02 ^b^	0.32 ± 0.01 ^b^	0.31 ± 0.01 ^b^	1.45 ± 0.01 ^c^	0.48 ± 0.04 ^a^
UD200	1.78 ± 0.03 ^b^	1.76 ± 0.01 ^c^	0.32 ± 0.01 ^b^	0.33 ± 0.01 ^b^	1.75 ± 0.01 ^a^	0.47 ± 0.02 ^a^
BPA + UD100	1.86 ± 0.05 ^a^	1.86 ± 0.04 ^a^	0.37 ± 0.01 ^a^	0.31 ± 0.01 ^b^	1.67 ± 0.01 ^ab^	0.48 ± 0.05 ^a^
BPA + UD200	1.77 ± 0.04 ^b^	1.81 ± 0.04 ^b^	0.37 ± 0.01 ^a^	0.36 ± 0.02 ^a^	1.55 ± 0.06 ^b^	0.46 ± 0.04 ^ab^

^a,b,c,d^: Different letters in the same column indicate statistically significant difference (*p* < 0.05). Data are presented as mean ± SD based on measurements obtained from eight animals per group (n = 8). BPA, bisphenol A; UD, *Urtica dioica*; GSI, gonadosomatic index.

**Table 2:** Effect of BPA and UD on spermatological parameters.

Groups	Sperm Concentration (×10^6^ cells/mL)	Total Sperm Motility (%)	Sperm Abnormalities (%)	Dead Spermatozoa (%)	HOST (%) (Membrane Integrity)
Control	198.375 ± 20.696	55.000 ± 2.520 ^b^	7.250 ± 0.495 ^c^	20.000 ± 2.444 ^b^	60.000 ± 3.490 ^c^
BPA	148.228 ± 14.100	46.428 ± 2.100 ^c^	18.575 ± 1.480 ^a^	35.250 ± 1.195 ^a^	53.000 ± 2.480 ^d^
UD100	200.275 ± 12.390	60.625 ± 3.530 ^ab^	6.200 ± 0.560 ^cd^	15.600 ± 2.380 ^bc^	72.000 ± 3.596 ^b^
UD200	180.200 ± 20.142	65.000 ± 3.580 ^a^	6.300 ± 0.254 ^cd^	10.000 ± 2.175 ^c^	75.400 ± 3.262 ^a^
BPA + UD100	216.356 ± 15.523	65.000 ± 3.382 ^a^	9.755 ± 0.500 ^b^	15.725 ± 2.156 ^bc^	73.000 ± 2.060 ^a^
BPA + UD200	220.225 ± 12.412	70.571 ± 2.102 ^a^	8.826 ± 0.750 ^bc^	10.250 ± 1.102 ^c^	80.000 ± 1.424 ^a^

^a,b,c,d^: Different letters in the same column indicate statistically significant difference (*p* < 0.05). Data are presented as mean ± SD based on measurements obtained from eight animals per group (n = 8). BPA, bisphenol A; UD, *Urtica dioica*; HOST, hypo-osmotic swelling test.

**Table 3:** UHPLC-Orbitrap^®^-HRMS phenolic compound profiling of UD ethanolic extract.

No	Target Compounds	RT	Quan Peak (*m*/*z*)	Curve Type	UD (µg/g of Extract)
1	Chlorogenic acid	7.73	353.08781	Linear	1216.356
2	Chrysin	13.54	253.05063	Linear	0.047
3	Esculin hydrate	7.05	339.07216	Quadratic	2.218
4	Luteolin-7-rutinoside	10.19	593.15119	Linear	173.883
5	Protocatechuic acid	5.23	109.02992	Linear	48.361
6	Quercetin	11.71	301.03538	Linear	5.625
7	Quinic acid	0.85	191.05611	Linear	6807.55
8	Rosmarinic acid	10.31	359.07724	Linear	264.42
9	Schaftoside	9.49	563.14063	Quadratic	41.533
10	Vicenin-2	8.86	593.15119	Quadratic	22.093
11	Vitamin C	0.87	175.02478	Quadratic	2.184

UHPLC-Orbitrap^®^-HRMS, ultra-high-performance liquid chromatography–Orbitrap^®^ high-resolution mass spectrometry; UD, *Urtica dioica*; RT, retention times.

**Table 4:** Semi-quantitative histopathological injury scores in testicular tissues of control and experimental groups.

Groups	Tubular Degeneration	Germ Cell Loss	Vacuolization	Pyknotic Nuclei	Interstitial Fibrosis/Edema	Total Injury Score
Control	0 (0–1)	0 (0–1)	0 (0–1)	0 (0–1)	0 (0–1)	1 (0–3) ^c^
BPA	3 (2–3)	3 (2–3)	2 (2–3)	3 (2–3)	2 (1–3)	13 (11–15) ^a^
UD100	0 (0–1)	0 (0–1)	0 (0–1)	0 (0–1)	0 (0–1)	1 (0–3) ^c^
UD200	0 (0–1)	0 (0–1)	0 (0–1)	0 (0–1)	0 (0–1)	0 (0–2) ^c^
BPA + UD100	1 (1–2)	2 (1–2)	1 (1–2)	1 (1–2)	1 (0–2)	7 (5–9) ^b^
BPA + UD200	1 (0–1)	1 (0–1)	0 (0–1)	1 (0–1)	0 (0–1)	3 (1–5) ^c^

Data are presented as median (minimum–maximum). Statistical comparisons were performed using the Kruskal–Wallis test followed by Dunn-Bonferroni multiple comparison test. Different superscript letters within the same column indicate statistically significant differences (*p* < 0.05). BPA, bisphenol A; UD, *Urtica dioica*.

## Data Availability

The original contributions presented in this study are included in the article/Supplementary Material. Further inquiries can be directed to the corresponding author.

## Supplementary Materials

The following supporting information can be downloaded at: https://www.mdpi.com/article/10.3390/vetsci13080840/s1, Original Western blot images.

## Abbreviations

BPA	Bisphenol A
ROS	Reactive oxygen species
UD	*Urtica dioica*
TPC	Total phenolic content
Na_2_CO_3_	Sodium carbonate
UV-Vis	Ultraviolet-visible
GAE	Gallic acid equivalents
UHPLC	Ultra-high performance liquid chromatography
HRMS	High-resolution mass spectrometry
HESI	Heated electrospray ionization
AGC	Automatic gain control
WHO	World Health Organization
HTF	Human tubal fluid
HOS	Hypo-osmotic swelling
H&E	Hematoxylin and eosin
PBS	Phosphate buffered saline
EDTA	Ethylenediaminetetraacetic acid
H_2_O_2_	Hydrogen peroxide
Bax	Bcl-2-associated X protein
Bcl-2	B-cell lymphoma 2
TNF-α	Tumor necrosis factor alpha
IL-1ß	Interleukin-1 beta
IL-6	Interleukin-6
DAB	3,3′-diaminobenzidine
RIPA	Radioimmunoprecipitation assay
BCA	Bicinchoninic acid
PVDF	Polyvinylidene fluoride
ECL	Enhanced chemiluminescence
CAT	Catalase
GPx	Glutathione peroxidase
SOD	Superoxide dismutase
LOD	Limits of detection
LOQ	Limits of quantification
SD	Standard deviation
GSI	Gonadosomatic index
RT	Retention times
